# An Emerging Tick-Borne Disease of Humans Is Caused by a Subset of Strains with Conserved Genome Structure

**DOI:** 10.3390/pathogens2030544

**Published:** 2013-09-10

**Authors:** Anthony F. Barbet, Basima Al-Khedery, Snorre Stuen, Erik G. Granquist, Roderick F. Felsheim, Ulrike G. Munderloh

**Affiliations:** 1 Department of Infectious Diseases and Pathology, University of Florida, Gainesville, FL 32611, USA; E-Mail: balkhedery@ufl.edu; 2 Emerging Pathogens Institute, University of Florida, Gainesville, FL 32611, USA; 3 Department of Production Animal Clinical Sciences, Norwegian School of Veterinary Science, Sandnes N-4325, Norway; E-Mail: snorre.stuen@nvh.no; 4 Department of Production Animal Clinical Sciences, Norwegian School of Veterinary Science, Oslo N-0033, Norway; E-Mail: erikgeorg.granquist@nvh.no; 5 Department of Entomology, University of Minnesota, St. Paul, MN 55108, USA; E-Mails: felsh001@umn.edu (R.F.F.); munde001@umn.edu (U.G.M.)

**Keywords:** anaplasmosis, tick-borne diseases, high-throughput sequencing, pfam01617, msp2/p44, comparative genomics

## Abstract

The prevalence of tick-borne diseases is increasing worldwide. One such emerging disease is human anaplasmosis. The causative organism, *Anaplasma phagocytophilum*, is known to infect multiple animal species and cause human fatalities in the U.S., Europe and Asia. Although long known to infect ruminants, it is unclear why there are increasing numbers of human infections. We analyzed the genome sequences of strains infecting humans, animals and ticks from diverse geographic locations. Despite extensive variability amongst these strains, those infecting humans had conserved genome structure including the pfam01617 superfamily that encodes the major, neutralization-sensitive, surface antigen. These data provide potential targets to identify human-infective strains and have significance for understanding the selective pressures that lead to emergence of disease in new species.

## 1. Introduction

Human exposure to vectors carrying disease agents has been increased by climate and land-use changes causing more contact between humans and domestic animals with wildlife reservoirs [1]. One such recently emerging disease is tick-borne anaplasmosis that causes infections in multiple animal species [2]. These include cattle, sheep, goats, horses, dogs, foxes, cats, rodents and most recently, humans. Different strains of the causative organism, *Anaplasma phagocytophilum*, have different host predilections and not all strains can infect all hosts. Small mammals are thought to be a reservoir, infecting immature stages of ticks which act as bridge vectors transferring infection to humans and domestic animals. Cases reported to the U.S. Centers for Disease Control and Prevention comprise a flu-like febrile illness with some severe sequelae such as multiple organ failure, or severe acute respiratory distress syndrome. In the U.S., case reports increased from 348 cases in 2000 to 1,761 cases in 2010 [3]. The reported hospitalization rate is 36% [4]. Granulocytic anaplasmosis (GA) can be treated with antibiotics, but the symptoms, such as headache, fever, and muscle aches are non-specific and can be confused with other common diseases such as Lyme, transmitted by the same species of tick. If left untreated GA can be severe [5], resulting in a case fatality rate in the U.S. of up to 3% (CDC data for 2003). Increasingly, there are reports of infections transmitted by blood transfusions in the U.S. and Europe [6,7]. Here we show that, in contrast to the extensive worldwide genomic diversity of *A. phagocytophilum* strains, human-infective strains are a conserved subset. This has implications for understanding the selective pressures that lead to emergence of disease in new species and for control of this infection.

## 2. Results and Discussion

### 2.1. Comparative Genomics of Nine Strains of A. phagocytophilum

**Figure 1:**
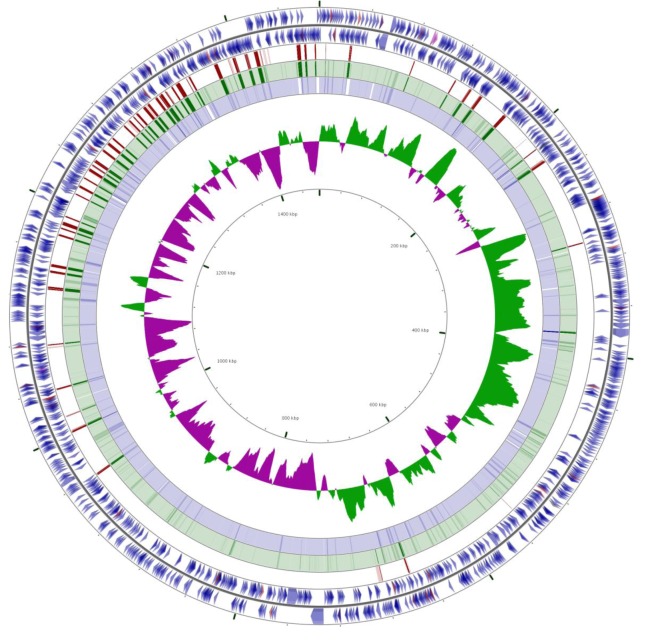
Genome synteny between two strains of *A. phagocytophilum*, ApHZ (human origin, NY, USA) and ApJM (rodent origin, MN, USA). The reference strain is ApHZ (GenBank CP000235). The two outermost circles show annotated ApHZ coding sequences, the third circle shows the locations of members of the ApHZ pfam01617 superfamily, the fourth and fifth circles are Blastn comparisons of the ApJM genome with ApHZ using 90% and 99% identity cutoffs respectively, the sixth circle shows GC skew and the innermost circle the numeric genome position. Comparisons were conducted using the CGView server using partial opacity to visualize overlapping hits (darker bars in the green and blue circles). The white bars on the fifth circle indicate no match.

Underlying the ecological complexity and variable host tropism of *A. phagocytophilum* is a large degree of genetic variability. European strains of *A. phagocytophilum* are highly pathogenic in sheep and cattle, whereas North American strains do not cause disease in domestic ruminants. Some U.S. strains, defined as Ap-variant 1 because of a 2 base pair difference in 16S rRNA, are infectious to deer and other ruminants, but do not infect mice and are thought not to infect humans [8,9]. Highly unusual for an obligate intracellular pathogen with a small, 1.5 Mb genome, *A. phagocytophilum* contains approximately 100 “functional pseudogenes” of the major surface protein 2 gene (*msp*2/*p44*) that codes for the major antigen on its surface [10]. These hypervariable pseudogene cassettes are recombined into a single expression site which provides *N*- and *C*-terminal conserved sequences, allowing the bacterium to serially express variable antigens and evade host immunity [11,12]. We applied high-throughput genome sequencing to nine strains, derived from humans and different host animal species from the U.S. and Europe. These strains are: two human origin strains from New York and Minnesota, a rodent- and dog-origin strain from Minnesota, two Ap-variant 1 strains from Minnesota, a horse-origin strain from California and two sheep-origin strains from Norway, for which no *in vitro* cultures are available. In Figure 1 the complete genome sequence of a strain derived from rodents in an area of high prevalence for human disease (Camp Ripley, MN, USA) is compared with that of the human ApHZ strain (from the state of New York). The figure shows Blast comparisons of the rodent and human strains, particularly the positions of members of the pfam01617 superfamily, to which *msp2/p44* belongs. Overall, despite widely separated geographic and species origins, the two genomes are conserved and almost completely syntenic. The majority of the highly repetitive *msp2/p44* genes (visualized using partial opacity) are located in the third of the genome closest to the origin of replication (top of figure, changing sign of GC skew), as reported previously [13]. However, using high-stringency Blast comparisons (fifth circle from the outside) differences between the two genomes were primarily localized to this region also and to *msp2/p44* genes in particular, showing the propensity of this gene family to undergo rapid diversification.

**Figure 2:**
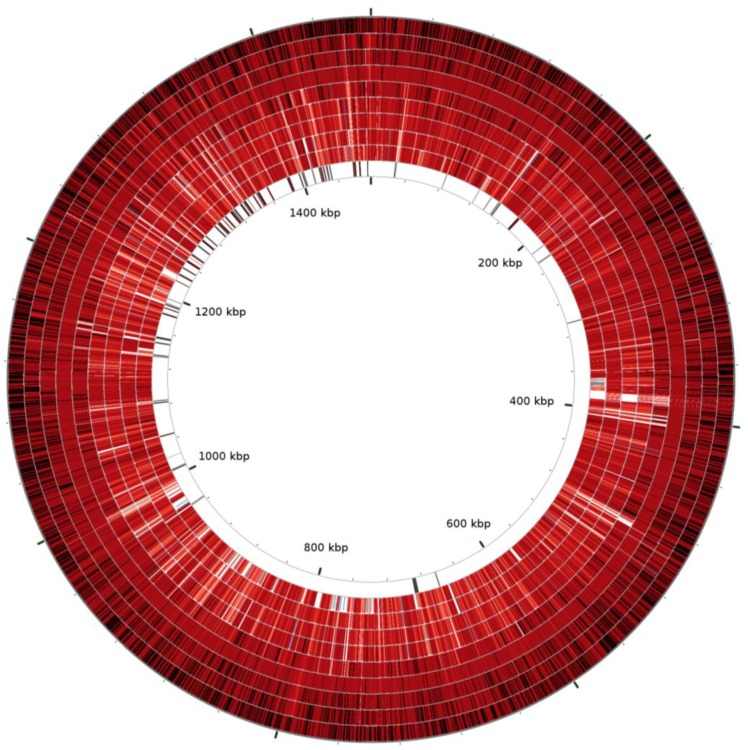
Similarities and differences between nine *A. phagocytophilum* genomes. Roche/454 reads from each genomic DNA were compared with the human ApHZ genome as reference using Blastn and the CGView Comparison Tool. The first to ninth circles compare, respectively, sequencing reads from ApHZ, ApDog, ApJM, ApHGE1, ApMRK, ApCRT35, ApCRT38-1, ApNorV2, ApNorV1 genomic DNAs. The 10th circle shows the locations of members of the ApHZ pfam01617 superfamily. Blast parameters used a query size of 500 bp segments of the reference genome and a Blast expect value of 10^−100^. Circles are colored according to the percent identities of matches (black to light red, 100%–90% identical, dark to light blue, 88%–82% identical, colorless, 0% identical).

This comparative genomics analysis was extended to all nine strains (Figure 2). Except for Ap-variant 1 strains, the U.S. strains had high identities across their entire genomes, with the ApDog strain most similar to human ApHZ by Blast analysis. The Norwegian sheep and U.S. Ap-variant 1 strains had the lowest identities with ApHZ in Blast comparisons, especially in members of the pfam01617 superfamily.

**Figure 3:**
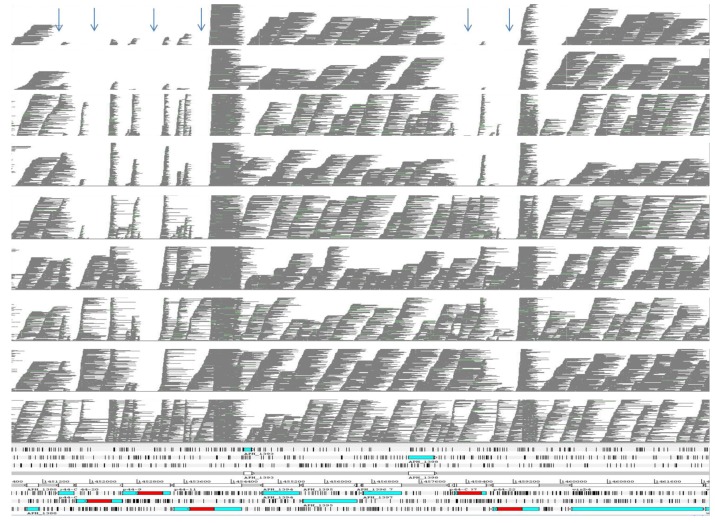
Gene variation in a region rich in *p44/msp2* pseudogenes close to the origin of replication in nine genomes of *A. phagocytophilum*. Roche/454 pyrosequencing reads from each of the nine genomes are shown aligned with the annotated reference ApHZ genome (bottom) between positions 1,450 and 1,462 kbp. The sequencing reads are derived from: ApHZ, ApDog, ApHGE1, ApJM, ApMRK, ApCRT35, ApCRT38-1, ApNorV1, ApNorV2 (bottom to top panels respectively). The positions of six *msp2/p44* pseudogenes are marked by arrows at the top and shown in their respective reading frames at the bottom. The hypervariable regions LAKT.. LAKT, present in five of the six pseudogenes, are indicated in red. Gaps in coverage indicate no aligning reads over the central hypervariable regions in some strains.

The differences in this superfamily were examined more closely in order to determine their *msp2/p44* genomic repertoires and if differences were primarily located in the known pseudogene hypervariable regions (Figure 3).

Similar to the rodent strain ApJM, the *msp2/p44* repertoires of a U.S. human strain, ApHGE1 and the ApDog strain from the same area of Minnesota were closely related and slightly different from the New York human strain ApHZ (8/95 or 9/95 different *msp2/p44* pseudogenes determined as <90% identical, Table 1). In contrast, strains infecting horses or ruminants (Ap-variant 1 strains) in the U.S. or Norway (representing “classical” strains long known to be pathogenic to sheep [14] shared fewer *msp2/p44* pseudogenes with human strains, with the two Norwegian sheep strains having almost totally different repertoires from human strains. Differences between individual members of the *msp2/p44* repertoire were located in the known central hypervariable region between flanking consensus amino acids LAKT.. LAKT (Figure 3). 

Table 1Genome structural relationships between *Anaplasma* strains.

**Table:** 

Strain	ApHZ	ApHGE1	ApJM	ApDog	ApMRK	ApCRT35	ApCRT38	ApNorV1	ApNorV2
Different *msp2/p44* pseudogenes from ApHZ	0/95	8/95	8/95	9/95	49/95	71/95	75/95	92/95	89/95
ANIm	100	98.81	98.84	98.79	97.76	96.28	96.21	94.92	95.87
Tetra	1.000	0.999	0.999	0.999	0.998	0.996	0.996	0.995	0.996

**Table:** 

Strain	AmFL	AmStM	AcIs	ApHZ
ANIm	100	98.86	88.89	77.82
Tetra	1.000	0.999	0.989	0.821

The top panel compares strains with ApHZ, the lower panel compares strains and species with AmFL. Abbreviations: ANIm, Average % genome nucleotide identity using Mummer; Tetra, correlation coefficient of tetranucleotide signature frequencies; AmFL, *Anaplasma marginale* Florida strain; AmStM, *A. marginale* St. Maries Idaho strain; AcIs, *Anaplasma centrale* Israel strain; Ap, *A. phagocytophilum* strains.

Two additional quantitative measures of genome structural relationships compare the average nucleotide identities or the tetranucleotide frequencies between two genomes. The latter method is independent of alignment algorithms. The proposed threshold for prokaryotic species separation is 94% for average nucleotide identity and 0.990 for the correlation coefficient of tetranucleotide signature frequencies [15]. It is evident from Table 1 that the existing taxonomy of *Anaplasma* species meets these definitions. However, it is apparent that by these measures also, the U.S. human, dog and rodent strains are closely related (average nucleotide identity 98.79%–98.84% with ApHZ). The Norwegian ruminant strains are again most distinct from ApHZ (average nucleotide identity 94.92%–95.87%), agreeing with the similar divergence observed in their pfam01617 superfamily genes.

### 2.2. Comparison of the msp2/p44 Family among Strains of A. phagocytophilum

Although these data showed that two U.S. *A. phagocytophilum* strains infecting humans, as well as a dog and rodent strain were similar to one another and different from strains infecting ruminants, we wanted to analyze this more globally using a larger dataset. To do this, we took advantage of the fact that conserved regions of *msp2/p44* have been used as sensitive PCR diagnostic targets because of the large copy number of the pfam01617 superfamily. More than 500 partial pseudogene sequences are present in GenBank, from humans, ticks and animal strains derived from multiple regions throughout the U.S., Europe and Asia. From these sequences it is possible to extract the hypervariable region of *msp2/p44* flanked by sequence encoding a consensus LAKT on either end, facilitating alignment [16]. Importantly, for this analysis it is necessary to recognize that multiple different hypervariable regions exist in a single genome because of the ~100 distinct pseudogene cassettes present. Therefore, it is not sufficient to simply analyze phylogenetic trees and apparent evolutionary relationships between all *msp2/p44* sequences. If one wishes to compare global repertoires with the genome-sequenced human ApHZ strain, it is necessary to align each *msp2/p44* sequence with all ~100 ApHZ strain genomic pseudogene cassettes to find the best fit (maximum sequence identity). If strains are related to ApHZ, one expects to find one or more ApHZ pseudogenes with high identity to other strain *msp2/p44*s. Our analyses, therefore, employed a matrix where every available *msp2/p44* polypeptide (661 total including those encoded by ApHZ pseudogene cassettes) was aligned with every other, generating 218,791 alignments having a mean amino acid sequence identity of 48.2%. These percentage sequence identities can be rapidly analyzed using a 661 (row) × 661 (column) spreadsheet. Surprisingly, despite the differences in *msp2/p44* repertoires observed previously, all 91 available *msp2/p44* human-origin sequences from widely dispersed U.S. locations (states of New York, Massachusetts, Wisconsin and Minnesota) encoded polypeptides with a mean maximum percentage identity with ApHZ of 97.2% (Table 2). Further, in analyzing the available *msp2/p44* sequences it was notable that, world-wide, other human-origin strains also achieved similar levels of sequence identity with ApHZ (e.g., 98.1% mean maximum percentage amino acid identity of a dataset from Japan comprising 27 human-origin *msp2/p44* variants, no significant difference with ApHZ). In contrast, *A. phagocytophilum* strains from Europe and Asia from non-human sources had mean maximum identities of <76% (significantly different from ApHZ, Table 2).

**Table 2:** Comparison of *msp2/p44* variants from different geographic locations with the human ApHZ strain.

Country	Source	Number of *msp2/p44* Variants Analyzed	Mean Maximum % a.a. Identity with ApHZ (+/−Std.Dev.)	Significantly different from U.S. Human *
U.S.A.	Human	91	97.2 (7.0)	
U.S.A.	Dog	27	**94.8 (9.8)**	**No**
U.S.A.	Horse	29	**94.2 (7.3)**	**No**
U.S.A.	Bear	4	92.4 (4.1)	N/A
U.S.A.	Woodrat	88	78.4 (13.8)	Yes
U.S.A.	Ruminant, tick (Ap-variant 1)	34	86.5 (14.7)	Yes
Norway	Sheep	54	66.6 (6.4)	Yes
Sweden	Dog	19	64.3 (7.9)	Yes
U.K.	Goat	20	67.2 (8.2)	Yes
U.K.	Sheep	19	65.1 (6.7)	Yes
Czech Republic	Human, Roe deer, Perdix, *Ixodes ricinus*	6	86.6 (14.5)	N/A
	[Human only]	[2]	[97.8][(2.0)]	N/A
Japan	Sika deer	17	39.3 (3.0)	Yes
Japan	*Ixodes persulcatus*	87	69.0 (6.9)	Yes
Japan	*Ixodes ovatus*	22	71.8 (15.5)	Yes
Japan	*Haemaphysalis formosensis*	9	75.6 (14)	N/A
Japan	Human	27	**98.1 (7.2)**	**No**
China	Human	2	100 (0)	N/A

* Kruskal-Wallis One Way Analysis of Variance on Ranks (*p* ≤ 0.001) followed by Dunn’s method for Multiple Comparisons *versus* a Control group (*p* ≤ 0.05). N/A: Not applied to sample sizes <10.

## 3. Experimental

### 3.1. Origin of A. phagocytophilum Strains

The origins of the *A. phagocytophilum* strains used in this study are as follows: ApHZ, human, Westchester County, New York; HGE1, human, Minnesota; ApJM, meadow jumping mouse (*Zapus hudsonius*), Camp Ripley, Minnesota; ApDog, Minnesota; ApCRT35, Camp Ripley tick (*Ixodes scapularis*), Minnesota (defined previously as Ap-variant 1); ApCRT38-1, Camp Ripley tick (*I. scapularis*), Minnesota (Ap-variant 1); ApMRK, horse, California; ApNorV1, sheep, Norway; ApNorV2, sheep, Norway. *A. phagocytophilum* genomic DNA was prepared from *in vitro* cultured organisms or directly from infected sheep (Norwegian strains), as described previously [17].

### 3.2. Ethics Statement

The experimental study in sheep was approved by the Norwegian Animal Research Authority.

### 3.3. Genome Sequencing and Bioinformatics

Genomic DNA was sequenced on the Roche/454 Genome Sequencer using non-paired and 3 kb paired-end libraries, also as described [17]. Mean genome coverage with respect to ApHZ varied between 31.3X and 72.1X. The ApJM sequence was finished using manual inspection for conflicts and mismatched paired ends and PCR to fill gaps. All sequences were compared with ApHZ for regions of identity using Blastn analysis in CGView [18,19]. Differences in the *msp2/p44* repertoires between strains were defined using a method previously validated using the pfam01617 superfamily and two completely Sanger-sequenced genomes of *Anaplasma marginale* [20]. Briefly, this method uses Mosaik to align individual reads and generate BAM format files to detect gaps in alignment (no coverage) with respect to the annotated reference sequence. In *A. marginale*, this method detected all different pseudogenes having <90% nucleotide identity with the reference genome. In homologous comparisons between ApHZ Roche/454 reads and the ApHZ genome all *msp2/p44* pseudogenes were detected as present (Table 1). Comparisons of average nucleotide identities and the correlation coefficients of tetranucleotide signature frequencies between genomes were conducted using Jspecies software, as described [15].

### 3.4. Analysis of msp2/p44 Repertoires

To analyze global *msp2/p44* repertoires, deposited *msp2/p44* sequences were downloaded from GenBank following Blast searches; several large datasets are also available from published studies [16,21,22,23,24,25]. The sequences were each trimmed to that encoding the hypervariable region, where possible using the consensus flanking LAKT residues, and the polypeptides were aligned (all against all) with MATGAT [26]. This generates the alignments that can be inspected for accuracy, and allows export of all percent identity values to a spreadsheet. This spreadsheet was analyzed in Excel for the relatedness of the different datasets. The percent identity of each variant *msp2/p44* sequence with every ApHZ *msp2/p44* polypeptide encoded by a pseudogene was reported and, from that, the mean best match with ApHZ (maximum percent identity) determined for each dataset. As the percentage identities were not normally distributed and the population variances were unequal, significant differences between groups were determined by nonparametric methods (Table 2).

## 4. Conclusions

These data show that there is genome diversity worldwide within the *A. phagocytophilum* species that extends close to some proposed guidelines for species discrimination. However, strains infecting humans are a subset with more conserved genome structure than the species overall, and this includes their repertoires of *msp2/p44* genes. This subset of *A. phagocytophilum* strains does not differ significantly from the U.S. ApHZ strain and is closely related to strains infecting U.S. domestic dogs. The mean seroprevalence (using a conserved *msp2/p44* peptide as antigen) in the U.S. among 479,640 dogs was 4.8% with prevalence >50% in some counties in the Northeast and Midwest, corresponding to the location of the majority of human cases [27]. These data have significance in several areas. First, *msp2/p44* encodes a protein that induces neutralizing antibodies against homologous strains of *A. phagocytophilum* [28,29]. The repertoire differences between strains reflect an evolving antigenic system with adaptive pressures imposed by an array of different persistently infected hosts. In contrast, humans are incidental dead-end hosts that do not impose significant pressure for change. The similarities between human-origin strains in the U.S., Europe and Asia suggest that humans may not be susceptible to many of the circulating wildlife strains. They may become susceptible when selection pressures in small mammal reservoir hosts cause evolution of novel strains that allow invasion and survival in humans. Rodents are important reservoirs for a multitude of human disease agents, vector-borne or not, and are known to be infected with strains of *A. phagocytophilum* having different species tropisms [30]. Rodent control has historically been emphasized as a means to control human disease outbreaks. The underlying evolutionary drivers remain to be identified but could reflect the long-standing association between “men and mice”. Second, these data have practical significance for control of this emerging infection. Despite the ubiquity of *A. phagocytophilum* strains and positive serology world-wide, it is necessary to focus on the prevalence and transmission of a smaller, human-infective subset. Differentiation of such strains from the high-prevalence background may be possible using genomic PCR targets or selected *msp2/p44* polypeptides for serology. In order to accomplish this goal it is necessary to acquire genome sequences from multiple human-origin strains on different continents. The technology now exists to do this.
